# Factors affecting caregiver burden in families of critically ill obstetric patients admitted to intensive care unit of a tertiary care hospital—a questionnaire based prospective cross-sectional observational study

**DOI:** 10.3389/fmed.2025.1706346

**Published:** 2025-12-15

**Authors:** Xinhong Guo, Yaqiong Jiang, Menghua Zhang

**Affiliations:** 1 Department of Gynecology, Nantong First People's Hospital, Nantong, China; 2 Department of Intensive Care Unit, Nantong First People's Hospital, Nantong, China

**Keywords:** critically ill obstetric patients, family caregivers, caregiver burden, influencing factors, multivariate linear regression analysis

## Abstract

**Aim:**

Family caregivers play a crucial role in the care of critically ill obstetric patients, yet their burden often goes unrecognized and unsupported. Despite the growing recognition of the importance of family caregivers in healthcare, there remains a significant knowledge gap regarding the specific burden they face and the factors influencing it. This study aimed to analyze the current situation of family caregivers' burden for critically ill obstetric patients and identify the related influencing factors.

**Methods:**

This study was conducted in a hospital setting, employing a cross-sectional design. A total of 150 family caregivers of critically ill obstetric patients who received treatment between January 2023 and December 2024 were selected using convenience sampling. Data were collected using the Zarit Caregiver Burden Interview (ZBI) to assess the burden of care and a self-designed general information questionnaire to gather demographic and relevant information. The primary outcome measure was the ZBI score, reflecting the level of burden experienced by family caregivers.

**Results:**

The mean ZBI score of the family caregivers was 45.15 ± 6.23 points, indicating a substantial burden of care. Multivariable linear regression analysis revealed that the length of stay for the patient in the hospital, as well as the age, education degree, family monthly income, social support level, and psychological resilience of family caregivers, were significantly associated with the burden of care (*P* < 0.05). Furthermore, Pearson correlation analysis demonstrated significant relationships between caregiver burden and the social support received by the family, as well as the psychological resilience of the family caregivers (*r* = −0.366, *P* < 0.001 and *r* = −0.307, *P* = 0.000).

**Conclusion:**

This study highlights the considerable burden faced by family caregivers of critically ill obstetric patients and identifies several key factors influencing this burden. The findings underscore the need for targeted interventions to support family caregivers, particularly those with lower social support and psychological resilience, to alleviate their burden and improve their well-being.

## Introduction

Caregiver burden is a critical concept in healthcare, referring to the difficulties, problems, or adverse effects that illness imposes on family caregivers (1). Clausen (1950s) first defined caregiver burden as the challenges and negative impacts experienced by those providing care to ailing family members (2). Kazemi et al. (3) further elaborated, describing caregiver burden as the extent to which caregivers' emotions, physical health, social life, and economic situation are affected by their caregiving responsibilities. Research has increasingly highlighted the magnitude of this problem, with studies showing that caregivers of patients with chronic conditions such as Alzheimer's disease, stroke, and cancer often face significant physical, psychological, economic, social, and emotional burdens (4–7). These burdens not only affect caregivers' well-being but also have implications for patients' recovery and quality of life (8).

Critically ill obstetric patients represent a high-risk group during pregnancy, labor, and postpartum recovery, often requiring intensive care monitoring due to life-threatening complications such as postpartum hemorrhage, preeclampsia/eclampsia, and placental abruption (9). These conditions can lead to multiple organ failure or severe metabolic disorders, necessitating continuous life support treatment. Family caregivers, as the primary support system for these patients, face immense pressures related to the disease, economic constraints, and rehabilitation needs (10). The prolonged length of stay in the hospital and the demanding nature of caregiving responsibilities further exacerbate the burden on family caregivers.

While there is a substantial body of research on caregiver burden in various patient populations, studies specifically focusing on the burden of family caregivers for critically ill obstetric patients are scarce. Existing literature has identified several factors influencing caregiver burden, including the age and educational level of caregivers, daily care hours, and the health status of patients (11–13). However, these studies have primarily been conducted in non-obstetric settings, leaving a gap in understanding the unique challenges faced by caregivers in the context of critically ill obstetric patients.

Previous research in this area has been limited by several factors. Many studies have focused on specific patient populations, such as those with chronic diseases (14), and have not adequately addressed the unique circumstances of critically ill obstetric patients. Additionally, the generalizability of findings from these studies is often constrained by small sample sizes, limited geographic scope, and a lack of consideration for cultural and socioeconomic diversity (15). Furthermore, the analysis of influencing factors has sometimes been superficial, failing to capture the complex interplay between various determinants of caregiver burden (16).

Our study addresses these gaps by providing a comprehensive analysis of the current situation of family caregivers' burden for critically ill obstetric patients and the related influencing factors. By focusing on this specific patient population, our research offers unique insights into the challenges faced by caregivers in a high-stress, high-stakes environment.

The primary aim of our study was to analyze the burden experienced by family caregivers of critically ill obstetric patients and to identify the key factors influencing this burden.

## Methods

### Study setting and design

This was a cross-sectional study conducted in the Maternal Intensive Care Unit (MICU) of a tertiary-level hospital in Nantong. The study aimed to assess the burden of family caregivers of critically ill obstetric patients and identify its influencing factors. A total of 150 family caregivers of critically ill obstetric patients who received treatment between January 2023 and December 2024 were selected using convenience sampling.

Based on the previous study (17), the sample size calculation using the *G* ^*^ Power software. Considering a significance level (α) of 0.05, a statistical power (1–β) of 0.80, and a 10% dropout rate, a total of 150 patients and their families were included.

All patients and their family caregivers signed the informed consent. This research was approved by the hospital's ethics committee (2024KT141). The clinical trial registration was ChiCTR2200063443.

### Participants

Inclusion criteria for patients: (1) Age ≥ 18 years; (2) diagnosed as critically ill obstetric patients; (3) no other comorbid organic diseases unrelated to pregnancy.

Exclusion criteria for patients: (1) patients with pre-existing mental illnesses; (2) patients transferred to another hospital or withdrawn from the study during data collection.

Inclusion criteria for family caregivers: (1) direct relatives of the patient (e.g., spouse, parents); (2) the patient was admitted to the MICU and required continuous care; (3) voluntary participation in the study.

Exclusion criteria for family caregivers: (1) family caregivers with severe mental illness or cognitive impairment; (2) inability to complete the questionnaire or interview due to language barriers or physical limitations.

### Questionnaires used

A self-designed general information questionnaire was used to collect the general information of critically ill obstetric patients (age, parity status, medical payment, kind of disease, length of stay in the hospital, and educational degree) and their family caregivers (gender, age, relationship with the patient, educational degree, working status, and family monthly income).

Zarit caregiver burden interview (ZBI) (18): this scale was used to assess the care burden of family caregivers. We obtained permission to use this scale from the original authors. The ZBI consists of 22 items, divided into personal burden and role burden. The scoring standard is based on the Likert 5-point scale, with scores ranging from 0 (never) to 4 points (always) for each item. The total score range is between 0 and 88 points. The higher the score, the heavier the care burden.

Social support rating scale (SSRS) (19): this scale was employed to assess the social support level of family caregivers, and we had permission to use it. It consists of 3 dimensions: subjective support, objective support, and utilization of support, with a total of 10 questions. The maximum score ranges from 12 to 66 points. A total score of <23 indicates a low social support level, while a total score ≥23 indicates a medium-high social support level.

Connor-Davidson Resilience Scale (CD-RISC) (20): this scale was used to assess the psychological resilience of family caregivers. It consists of three dimensions: strength, tenacity, and optimism, with 25 questions in total. Each question is scored from 0 to 4, and the total score ranges from 0 to 100. A higher score indicates a higher level of psychological resilience. A total score <35 is considered poor, while a total score ≥35 is considered good.

The questionnaires were available in the local vernacular language (Chinese). Before use, the original English versions of the questionnaires were translated into Chinese by a professional translator. Then, a back-translation was carried out by another independent translator. The two versions were compared and discrepancies were resolved through discussion among a panel of experts in the relevant fields to ensure the accuracy and validity of the Chinese versions. The three questionnaires were included in the Supplementary material for the reader's reference.

### Procedure and methods

To ensure the reliability and comprehensiveness of the data, all the questionnaires in this study were collected by the research team who had undergone strict training. Before collecting the questionnaires, the team members received systematic training covering the questionnaire content, communication skills, and neutrality principles to minimize errors during the collection process.

For family caregivers who could independently fill out the questionnaire, the researchers first explained the filling method of the questionnaires to them in a gentle and patient manner, including the purpose of each part, the way to answer various questions, and the precautions during the filling process. At the same time, they clearly informed the respondents that they could raise questions at any time during the filling process, and the researchers would give clear and accurate answers promptly. After the respondents indicated that they understood and confirmed that they could independently fill out the questionnaire, they were provided with a quiet and independent space where they could complete the questionnaire filling by themselves, to ensure that they could express their thoughts and feelings freely without any interference.

Considering that some family caregivers were older, had a low educational level, or had other situations that prevented them from independently completing the questionnaires, the researchers adopted the methods of oral explanation and proxy filling. When explaining the questionnaire items to these respondents, the researchers used extremely clear and understandable language that was close to daily life and avoided using professional terms or complex sentences to ensure that the respondents could easily understand the meaning of each question. During the oral explanation, the researchers maintained a neutral attitude and did not give any hints or guidance, but simply objectively conveyed the information of the questions. Based on the respondents' answers, the researchers accurately and truthfully filled in the corresponding positions of the questionnaires. To avoid recording errors, after questionnaire filling was completed, the researchers would repeat the answers to the respondents and obtain their confirmation before continuing to ask and fill in the next question.

The research team formulated a standardized questionnaire collection process and a unified language expression template. All researchers must strictly follow this process and template when collecting questionnaires to ensure consistency in the way and content of communication with different respondents and to minimize subjective biases caused by individual expression differences. During the questionnaire collection process, a two-person collaboration model was adopted. One researcher was responsible for communicating with the respondents, explaining the questions and recording their answers, while the other researcher was responsible for supervision and recording the verification situation. When questions or inconsistencies occurred, the two would jointly reconfirm with the respondents to ensure the accuracy and objectivity of the information. After the questionnaire collection was completed, cross-checking was carried out to further verify whether there were subjective biases in the questionnaire filling process.

This study distributed 200 questionnaires, and 150 valid responses were recovered, with an effective rate of 95.3%. In these 150 valid questionnaires, there was no data missing.

### Statistical methods

Data were entered and analyzed using SPSS 22.0 software. For continuous variables, descriptive statistics were presented in the form of mean ± standard deviation; for categorical variables, they were presented in the form of numbers and percentages. The normality of the variables was evaluated using the Shapiro-Wilk test. Differences were assessed using the independent sample t-test or the one-way analysis of variance (ANOVA). Pearson correlation analysis was use to analyze the correlation between burden of care of family caregivers of critically ill obstetric patients and their levels of social support and psychological resilience. Multiple linear regression analysis was used to analyze the influencing factors of caregiver burden.

## Results

### General data of critically ill obstetric patients

A total of 150 critically ill obstetric patients were included in this study, aged from 20 to 40 years old, with the average age of 34.26 ± 4.87 years old. There were 63 primiparas and 87 multiparas in this study. The detailed information was shown in Table 1.

**Table 1:** General data of critically ill obstetric patients.

**Items**	**Number and percentage**
**Age**	
20–30 years	65 (43.33)
30–40 years	85 (46.67)
**Parity status**	
Primipara	63 (42.00)
Multipara	87 (58.00)
**Medical payment**	
Health insurance	90 (60.00)
Self-paying	60 (40.00)
**Kind of disease**	
Postpartum hemorrhage	59 (39.33)
Preeclampsia/eclampsia	41 (27.33)
Placental abruption	50 (33.34)
**Length of stay in the hospital**	
<7 days	51 (34.00)
7–15 days	78 (52.00)
15–30 days	21 (14.00)
**Education degree**	
Primary school or junior high school	40 (26.67)
High school or vocational school	62 (41.33)
College degree or above	48 (32.00)

### General data of family caregivers of critically ill obstetric patients

A total of 150 family caregivers of critically ill obstetric patients were included in this study, aged from 23 to 60 years old, with the average age of 43.26 ± 5.28 years old. There were 80 males and 70 females in this study. The detailed information was shown in Table 2.

**Table 2:** General data of family caregivers of critically ill obstetric patients.

**Items**	**Number and percentage**
**Gender**	
Male	80 (53.33)
Female	70 (46.67)
**Age**	
<30 years	35 (23.33)
30–60 years	115 (76.67)
**Relationship with the patient**	
Spouse	73 (48.67)
Parents	77 (51.33)
**Education degree**	
Primary school or junior high school	45 (30.00)
High school or vocational school	60 (40.00)
College degree or above	45 (30.00)
**Working status**	
On-the-job	95 (63.33)
Not on-the-job	55 (36.67)
**Family monthly income**	
<3,000 yuan	57 (38.00)
≥3,000 yuan	93 (62.00)

### The burden of care for family caregivers of critically ill obstetric patients

The mean ZBI scores of the 150 family caregivers were 45.15 ± 6.23 points, suggesting that the family caregivers of critically ill obstetric patients usually bear a heavy burden of care.

### Correlation between burden of care of family caregivers of critically ill obstetric patients and their levels of social support and psychological resilience

**Figure 1:**
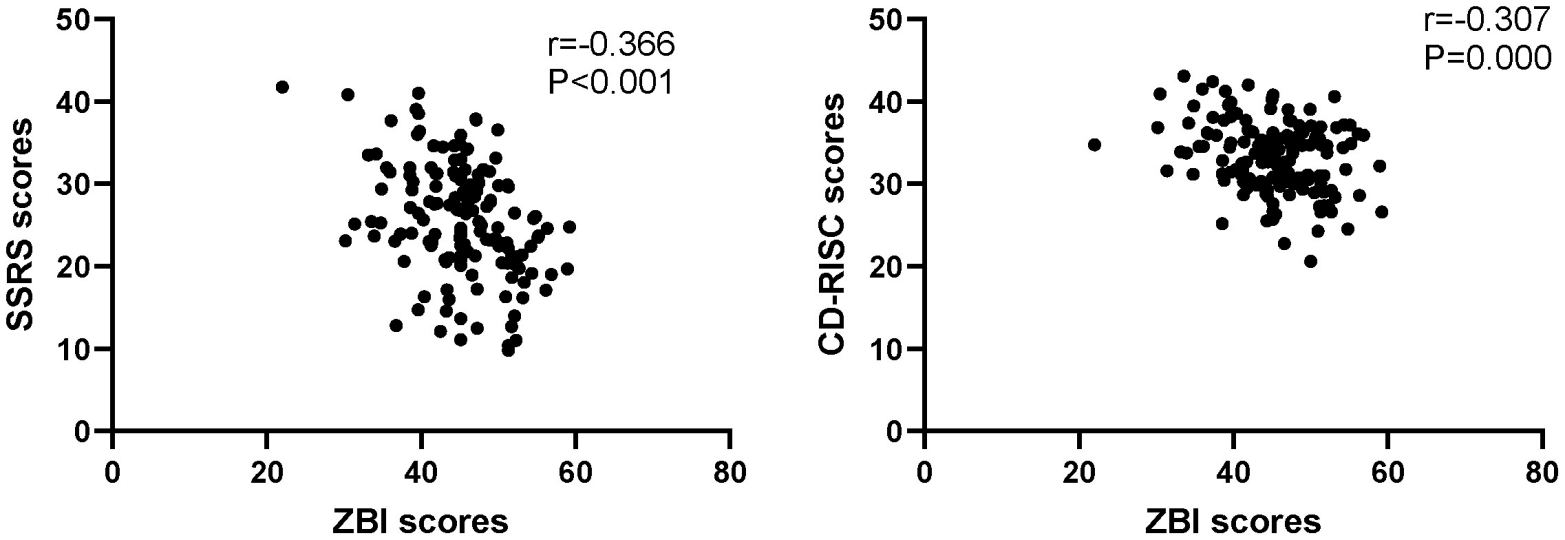
Pearson correlation analysis between ZBI scores and SSRS scores or CD-RISC scores.

The mean SSRS scores of the 150 family caregivers were 25.68 ± 6.87 points, and the mean CD-RISC scores of the 150 family caregivers were 33.27 ± 4.29 points. More importantly, Pearson correlation analysis revealed that the ZBI scores were negatively correlated with SSRS scores and CD-RISC scores (*r* = −0.366, *P* < 0.001 and *r* = −0.307, *P* = 0.000), as shown in Figure 1.

### Comparison of ZBI scores of critically ill obstetric patients

This study did not involve the patients themselves in the ZBI scoring process. The ZBI scoring was completed by the family caregivers of critically ill obstetric patients based on the actual basic information of the patients and their own true feelings and experiences during the care process.

As shown in Table 3, the burden of care for the family caregivers of critically ill obstetric patients showed statistically significant differences in terms of the patients' age and length of stay in the hospital (*P* < 0.001). There were no significant differences in the influence of the type of pregnant woman, medical payment, kind of disease and education degree (*P* > 0.05).

**Table 3:** Comparison of ZBI scores of critically ill obstetric patients.

**Items**	**Number and percentage**	**ZBI score**	***t*/*F* value**	***P* value**
**Age**				
20–30 years	65 (43.33)	40.25 ± 4.16	4.190	<0.001
30–40 years	85 (46.67)	43.25 ± 4.48		
**Parity status**				
Primipara	63 (42.00)	44.58 ± 4.58	0.969	0.334
Multipara	87 (58.00)	45.32 ± 4.64		
**Medical payment**				
Health insurance	90 (60.00)	45.09 ± 5.32	1.251	0.212
Self-paying	60 (40.00)	46.23 ± 5.68		
**Kind of disease**				
Postpartum hemorrhage	59 (39.33)	45.38 ± 4.62	1.726	0.181
Preeclampsia/ eclampsia	41 (27.33)	44.35 ± 4.57		
Placental abruption	50 (33.34)	43.78 ± 4.49		
**Length of stay in the hospital**				
<7 days	51 (34.00)	40.69 ± 4.05	22.118	<0.001
7–15 days	78 (52.00)	45.68 ± 4.67		
15–30 days	21 (14.00)	46.28 ± 4.74		
**Education degree**				
Primary school or junior high school	40 (26.67)	41.25 ± 4.26	1.046	0.353
High school or vocational school	62 (41.33)	42.08 ± 4.35		
College degree or above	48 (32.00)	42.59 ± 4.41		

### Comparison of ZBI scores of family caregivers of critically ill obstetric patients

As shown in Table 4, the burden of care for the family caregivers of critically ill obstetric patients showed statistically significant differences in terms of the family caregivers' age, education degree, family monthly income, social support level and psychological resilience (*P* < 0.001). There were no significant differences in the influence of gender, relationship with the patient and working status, (*P* > 0.05).

**Table 4:** Comparison of ZBI scores of family caregivers of critically ill obstetric patients.

**Factors**	***N***	**ZBI score**	***t*/*F* value**	***P* value**
**Gender**				
Male	80	46.08 ± 4.72	1.442	0.151
Female	70	44.98 ± 4.59		
**Age**				
<30 years	35 (23.33)	41.26 ± 4.38	5.085	<0.001
30–60 years	115 (76.67)	45.85 ± 4.76		
**Relationship with the patient**				
Spouse	73	45.31 ± 4.65	0.398	0.690
Parents	77	45.01 ± 4.56		
**Education degree**				
Primary school or junior high school	45	47.23 ± 4.75	15.159	<0.001
High school or vocational school	60	44.05 ± 4.39		
College degree or above	45	42.09 ± 4.31		
**Working status**				
On-the-job	95	45.60 ± 6.08	0.447	0.655
Not on-the-job	55	45.16 ± 5.29		
**Family monthly income**				
<3,000 yuan	57	46.85 ± 5.36	3.042	0.002
≥3,000 yuan	93	44.26 ± 4.87		
**Social support level**				
Low	50	45.02 ± 4.64	3.225	0.001
Medium-high	100	47.68 ± 4.82		
**Psychological resilience**				
Poor	99	46.85 ± 4.73	4.391	<0.001
Good	51	43.28 ± 4.69		

### Multivariate linear regression analysis of the influencing factors of the care burden of family caregivers of critically ill obstetric patients

The burden of care for the family caregivers of critically ill obstetric patients was taken as the dependent variable (a continuous variable), and the variables that were identified as significant after comparison in Tables 3, 4 were included as independent variables. Through multivariate linear regression analysis, the results demonstrated that the length of stay for the patient in the hospital, as well as the age, education degree, family monthly income, social support level and psychological resilience of family caregivers, were all factors influencing the family care burden (*P* < 0.05, Table 5).

**Table 5:** Multivariate linear regression analysis of the influencing factors of the care burden of family caregivers of critically ill obstetric patients.

**Independent variables**	***B* value**	**SE value**	**β value**	***T*-value**	***P*-value**
Constant	36.140	16.251		2.225	0.002
Age of patients	−0.810	1.390	−0.004	−0.060	0.948
Length of stay in the hospital	8.765	2.256	0.270	3.339	0.001
Age of family caregivers	1.458	1.520	0.150	1.068	0.025
Education degree	2.165	0.362	0.273	5.152	0.001
Family monthly income	2.156	2.583	0.215	6.621	0.001
Social support level	5.275	1.325	0.392	3.278	0.001
Psychological resilience	5.628	1.347	0.415	3.476	0.001

## Discussion

Our study analyzed the current care burden faced by family caregivers of critically ill obstetric patients, and found that the average ZBI score of 150 family caregivers was 45.15 ± 6.23 points, indicating that the family members of critically ill obstetric patients usually undertake heavy caregiving tasks. Consistently, an analysis conducted in a radiotherapy clinic in Nigeria regarding the care burden of family members of advanced breast cancer patients also showed that such family members were under considerable burden during the caregiving process (21).

The results of multivariate linear regression analysis indicated that the length of hospital stay for patients was an important factor influencing the care burden of family members of critically ill obstetric patients. This is because critically ill obstetric patients typically have longer hospital stays, requiring continuous monitoring of vital signs, invasive treatments, and handling of complications. The longer the hospital stay, the more time and energy the family caregivers need to invest, thereby increasing the burden. Parvizi et al. (22) indicated that the duration of caregiving is an important factor affecting the care burden of family members of cancer patients. This is consistent with the results of this study regarding the impact of hospital stay on the care burden of family members of critically ill obstetric patients, indicating that in various critical patient care scenarios, the time and energy consumption resulting from long-term care is a common factor causing the burden to increase for family members.

The results of multivariate linear regression analysis indicated that age was also a key variable influencing the burden of care. For family caregivers aged between 30 and 60, as they grow older, in addition to fulfilling the role of a caregivers, they also play significant roles in the family and society. Their physical condition may deteriorate, leading to an increase in the burden of care. Rahmani et al. (23) indicated that age, educational level, income, and disease duration of caregiving are significant predictors of the burden of care for patients with schizophrenia. This indicates that in different disease care scenarios, the decline in physical functions and the social role pressure experienced by family caregivers due to age growth will have a significant impact on the burden of care.

Furthermore, in this study, educational level was also proven to be an important factor influencing the caregiving burden of family members. Family members with higher cultural levels have a deeper understanding of the disease and pay more attention to the development of the patient's condition, as well as pay more attention to their own psychological adjustment. They actively seek information about disease recovery through various channels, are good at seeking external help, avoid psychological problems caused by uncertainty, and reduce the burden of care. However, family members with lower cultural levels have a stronger perception of the uncertainty of the patient's condition. They do not pay attention to their own mental health and do not know how to seek help from medical staff, which leads to more negative emotions and a more negative attitude toward future life, thereby increasing the burden of care. Surprisingly, in this study, it was found that some well-educated family members, despite having rich knowledge reserves, also experienced excessive anxiety and decision-making difficulties when facing the complex conditions and emergencies of critically ill obstetric patients. To a certain extent, this exacerbated their psychological burden, which was a negative result that was not fully anticipated before the study.

In addition, our study found that the monthly family income was also a key factor influencing the burden of care. Families with poor economic conditions not only have to deal with the stress brought by the patient's illness and its subsequent rehabilitation and exercise, but also bear huge economic costs, experience more negative emotions, and increase the burden of care. In line with our findings, Akter et al. (24) suggested that low-income levels are significantly associated with a higher caregiver burden for cancer patients. Economic pressure often forces family members to make difficult trade-offs when providing care for the patient, having to choose between medical expenses and living expenses. This undoubtedly increases their psychological burden. Surprisingly, some middle-income families also face economic pressure when dealing with the long-term treatment of critically ill obstetric patients. This is mainly due to the uncertainty of medical expenses and the existence of some self-funded items, which exceed their budgets and bring them considerable economic and psychological pressure.

At the same time, our study found that the social support level was also a key factor influencing the burden of care. According to the social support theory, if caregivers can receive understanding and support from others (especially relatives, friends, colleagues, and other people around them), then when they are affected and impacted due to caring for patients, they will feel that their efforts are meaningful and valuable. This helps them adapt to the changed lifestyle and thereby minimize the stress and burden of caregivers (25). According to the family systems theory and family dynamics theory, social support, as an external resource of the family, can help regulate the impact of stress events on the family. The more social support an individual receives, the better the adjustment effect will be (26). Consistently, Shokrgozar et al. proposed the correlation between the increase of social support and the reduction of the burden of caregivers for patients with bipolar disorder (27). In this study, it was also found that family members with higher level of social support could receive more practical assistance and psychological comfort when facing care problems, and their care burden was relatively lighter. However, surprisingly, some family members, despite receiving a considerable amount of social support, suddenly increased their care burden when this support was overly concentrated (such as relying entirely on relatives). This situation occurred when the relatives themselves encountered problems or had limited energy.

In addition, our study indicated that the psychological resilience of family caregivers was an important risk factor for the care burden of family caregivers of critically ill obstetric patients. Family caregivers with good psychological resilience know how to actively seek social support and assistance, which can enhance their caregiving skills, maintain a positive attitude toward caregiving behaviors, have confidence in their future life, reduce negative emotions, and alleviate the burden of care. However, family caregivers with poor psychological resilience are reluctant to communicate with others, have low caregiving efficiency, and thus increase the caregiving tasks, resulting in an increased burden. Similarly, Seo et al. (28) suggested that depression among family caregivers is an important factor affecting the caregiving burden of hospitalized lung cancer patients. In this study, it was also found that family members with poor psychological resilience were more likely to experience psychological problems, such as depression and anxiety, which in turn affected the quality of care. Surprisingly, some family members who originally seemed to have strong psychological adaptability experienced psychological breakdown when facing the long-term caregiving pressure of critically ill obstetric patients. This indicates that the maintenance and improvement of psychological adaptability require continuous attention and support.

## Strengths and limitations

This study possesses several distinctive strengths compared to previous related research. Unlike many prior studies that may have focused solely on either the clinical outcomes of critically ill obstetric patients or the general well-being of family caregivers without a comprehensive assessment tool, our study specifically employed the ZBI to systematically measure the burden of family caregivers of critically ill obstetric patients. This well-validated and widely-used scale provides a more in - depth and standardized understanding of the nature and magnitude of the caregiver burden, enabling us to obtain more precise and comparable data. Moreover, our study considered a relatively comprehensive set of factors that could potentially influence the caregiver burden. We not only examined patient-related factors but also explored caregiver-related factors. This multi-dimensional approach allows for a more holistic analysis of the factors contributing to the caregiver burden, which has not been fully addressed in some previous studies that may have only focused on a limited number of variables.

This study still has certain limitations. Firstly, as a cross-sectional survey, it is unable to analyze the trend of the family caregivers' burden of critically ill obstetric patients over time. To overcome this limitation, future research could adopt a longitudinal study design. By following up with the same group of family caregivers at multiple time points during the patient's illness and recovery process, we can gain a better understanding of how the caregiver burden evolves over time, identify critical periods when the burden is particularly high, and develop more timely and targeted interventions. Secondly, the sample size included in this study is limited. This may restrict the generalizability of our findings to a broader population. Further research needs to expand the sample size for investigation. A larger and more diverse sample that includes patients and caregivers from different regions, hospitals, and socio- economic backgrounds would increase the reliability and external validity of the results. Thirdly, this study adopts a questionnaire-based survey method, and the obtained results may be influenced by the subjective choices of family caregivers, thus resulting in certain bias in the results. To address this issue, future studies could combine multiple data collection methods. In addition, since only the caregivers of patients from the same hospital were selected, the representativeness of the sample was somewhat limited. Different hospitals may have differences in medical resources, diagnostic and treatment levels, and characteristics of the patient population. These differences may affect the stress conditions endured by the caregivers and the related influencing factors. Therefore, the results of this study may not be fully applicable to other hospitals of different levels and in different regions. Furthermore, convenience sampling may result in an imbalance in certain characteristics of the sample. We may be more likely to select family caregivers who are willing to cooperate with the research and have relatively more available time, while those family caregivers who are unwilling to participate for various reasons are excluded. This may cause the sample to have deviations in characteristics such as age, gender, educational level, and social support level compared to the overall population, thereby affecting the accuracy and reliability of the research results. To mitigate the impact of this limitation on the research results, in future studies, we will consider adopting methods such as multi-stage sampling or stratified sampling, and select samples from multiple hospitals at different levels and in different regions to enhance the representativeness and comprehensiveness of the samples. At the same time, during the research design stage, we will strengthen communication with potential research subjects and adopt more flexible and diverse methods to invite them to participate in the research, in order to minimize sample deviations caused by subjective factors.

The findings of this study have important clinical implications that can translate into better clinical practice. For healthcare providers, understanding the multi-faceted factors influencing the family caregiver burden is crucial. By identifying high-risk groups of caregivers, such as those with low social support, low psychological resilience, or low monthly family income, healthcare teams can provide targeted support and interventions. In addition, healthcare providers should pay more attention to the communication with family caregivers. Providing clear and timely information about the patient's condition, treatment plan, and prognosis can reduce caregivers' anxiety and uncertainty, thereby alleviating their burden. Moreover, involving family caregivers in the care planning process can make them feel more in control and valued, which can also contribute to a reduction in their burden.

## Conclusion

Our study reveals that family caregivers of critically ill obstetric patients experience a moderate level of burden. Notably, several factors significantly influence this burden, including the patient's length of hospital stay, along with the age, educational attainment, family monthly income, social support level, and psychological resilience of the family caregivers themselves. These findings underscore the complexity of the caregiver burden in this specific clinical context and highlight the need for tailored interventions to alleviate such burden.

## Data Availability

The original contributions presented in the study are included in the article/Supplementary material, further inquiries can be directed to the corresponding author.
