# Supplementary material for: Factors affecting caregiver burden in families of critically ill obstetric patients admitted to intensive care unit of a tertiary care hospital—a questionnaire based prospective cross-sectional observational study
Source: Front Med (Lausanne). 2025 Dec 15;12:1706346. doi: 10.3389/fmed.2025.1706346 (PMC12745222; doi:10.3389/fmed.2025.1706346)
Supplement: Supplementary file 1 [file Table_1.docx]

**Connor-Davidson Resilience Scale (CD-RISC)**

| Number | Questions | Options | | | | |
| --- | --- | --- | --- | --- | --- | --- |
|  |  | Never | Seldom | Sometimes | Often | Always |
| 1 | Able to adapt to change | 0 | 1 | 2 | 3 | 4 |
| 2 | Close and secure relationships | 0 | 1 | 2 | 3 | 4 |
| 3 | Sometimes fate or God can help | 0 | 1 | 2 | 3 | 4 |
| 4 | Can deal with whatever comes | 0 | 1 | 2 | 3 | 4 |
| 5 | Past success gives confidence for new challenge | 0 | 1 | 2 | 3 | 4 |
| 6 | See the humorous side of things | 0 | 1 | 2 | 3 | 4 |
| 7 | Coping with stress strengthens | 0 | 1 | 2 | 3 | 4 |
| 8 | Tend to bounce back after illness or hardship | 0 | 1 | 2 | 3 | 4 |
| 9 | Things happen for a reason | 0 | 1 | 2 | 3 | 4 |
| 10 | Best effort no matter what | 0 | 1 | 2 | 3 | 4 |
| 11 | You can achieve your goals | 0 | 1 | 2 | 3 | 4 |
| 12 | When things look hopeless, I don’t give up | 0 | 1 | 2 | 3 | 4 |
| 13 | Know where to turn for help | 0 | 1 | 2 | 3 | 4 |
| 14 | Under pressure, focus and think clearly | 0 | 1 | 2 | 3 | 4 |
| 15 | Prefer to take the lead in problem solving | 0 | 1 | 2 | 3 | 4 |
| 16 | Not easily discouraged by failure | 0 | 1 | 2 | 3 | 4 |
| 17 | Think of self as strong person | 0 | 1 | 2 | 3 | 4 |
| 18 | Make unpopular or difficult decisions | 0 | 1 | 2 | 3 | 4 |
| 19 | Can handle unpleasant feelings | 0 | 1 | 2 | 3 | 4 |
| 20 | Have to act on a hunch | 0 | 1 | 2 | 3 | 4 |
| 21 | Strong sense of purpose | 0 | 1 | 2 | 3 | 4 |
| 22 | In control of your life | 0 | 1 | 2 | 3 | 4 |
| 23 | I like challenges | 0 | 1 | 2 | 3 | 4 |
| 24 | You work to attain your goals | 0 | 1 | 2 | 3 | 4 |
| 25 | Pride in your achievements | 0 | 1 | 2 | 3 | 4 |
