# Supplementary material for: Factors affecting caregiver burden in families of critically ill obstetric patients admitted to intensive care unit of a tertiary care hospital—a questionnaire based prospective cross-sectional observational study
Source: Front Med (Lausanne). 2025 Dec 15;12:1706346. doi: 10.3389/fmed.2025.1706346 (PMC12745222; doi:10.3389/fmed.2025.1706346)
Supplement: Supplementary file 2 [file Table_2.docx]

**Social Support Rating Scale (****SSRS)**

1. How many close friends do you have who can offer support and assistance to you? (Please choose only one option)
   (1) None
   (2) 1-2
   (3) 3-5
   (4) 6 or more than 6

2. Over the past year, you: (please select one option)

(1) Have been away from your family and living alone in one room.

(2) Have frequently changed your residence and spent most of your time with strangers.

(3) Have been living with classmates, colleagues or friends.

(4) Have been living with your family.

3. Your relationship with neighbors: (Select only one option)

(1) You and your neighbors never care for each other; it's just a casual acquaintance.

(2) You might show some concern when facing difficulties.

(3) Some of your neighbors are very concerned about you.

(4) Most of your neighbors are very concerned about you.

4. Your relationship with colleagues: (Select only one option)

(1) You and your colleagues never care for each other; it's just a casual acquaintance.

(2) When facing difficulties, you might show a little concern.

(3) Some colleagues are quite concerned about you.

(4) Most colleagues are very concerned about you.

5. Support and care received from family members (Choose the appropriate option from the four options: none, little, moderate, full support)

A. Spouse (Partner) Parents

C. Children

D. Siblings

E. Other members (such as the sister-in-law)

6. In the past, when you encountered difficult situations, the sources of economic support and assistance in solving practical problems were as follows:

(1) No source.

(2) The following sources (multiple options are available):

A. Spouse

B. Other family members

C. Friends

D. Relatives

E. Colleagues

F. Work unit

G. Official or semi-official organizations such as political parties, trade unions, etc.

H. Non-governmental organizations such as religious and social groups

I. Others (please list)

7. In the past, when you encountered difficult situations, the sources of comfort and care you received were as follows:

(1) No source at all.

(2) The following sources (multiple options are available)

A. Spouse

B. Other family members

C. Friends

D. Relatives

E. Colleagues

F. Work unit

G. Official or semi-official organizations such as political parties, trade unions, etc.

H. Non-official organizations such as religious and social groups

I. Others (please list)

8. The way you express your troubles: (Select only one option)

(1) Never tell anyone.

(2) Only tell 1-2 very close friends.

(3) You will speak out if your friends ask you to.

(4) You will openly express your troubles to seek support and understanding.

9. The way you seek help when facing troubles: (Select only one option)

(1) Depend solely on yourself and refuse to accept help from others.

(2) Rarely ask for help from others.

(3) Sometimes ask for help from others.

(4) When in trouble, often seek assistance from family members, friends, or organizations.

10. Regarding activities organized by groups (such as political party organizations, religious organizations, trade unions, student unions, etc.), you: (please select only one option)

(1) Never participate

(2) Occasionally participate

(3) Frequently participate

(4) Actively participate and actively engage in the activities.
