# Supplementary material for: Factors affecting caregiver burden in families of critically ill obstetric patients admitted to intensive care unit of a tertiary care hospital—a questionnaire based prospective cross-sectional observational study
Source: Front Med (Lausanne). 2025 Dec 15;12:1706346. doi: 10.3389/fmed.2025.1706346 (PMC12745222; doi:10.3389/fmed.2025.1706346)
Supplement: Supplementary file 3 [file Table_3.docx]

**Zarit Caregiver Burden Interview (ZBI)**

| Items | Options | | | | |
| --- | --- | --- | --- | --- | --- |
|  | Never | Seldom | Sometimes | Often | Always |
| 1. Do you think that the patients you are taking care of might make too many demands for your assistance? | 0 | 1 | 2 | 3 | 4 |
| 2. Do you think that caring for patients will leave you with insufficient time for yourself? | 0 | 1 | 2 | 3 | 4 |
| 3. Do you think that you feel stressed when balancing the task of caring for patients with the responsibilities of household chores and work? | 0 | 1 | 2 | 3 | 4 |
| 4. Do you think you felt troubled by the patient's behavior? | 0 | 1 | 2 | 3 | 4 |
| 5. Do you think that having patients around you causes you any distress? | 0 | 1 | 2 | 3 | 4 |
| 6. Do you think that your patients have affected the relationships between you, your family members, and your friends? | 0 | 1 | 2 | 3 | 4 |
| 7. Are you worried about the future of the patient? | 0 | 1 | 2 | 3 | 4 |
| 8. Do you think that the patient relies on you? | 0 | 1 | 2 | 3 | 4 |
| 9. Do you feel nervous when the patient is by your side? | 0 | 1 | 2 | 3 | 4 |
| 10. Do you think that your health has been affected because of taking care of patients? | 0 | 1 | 2 | 3 | 4 |
| 11. Do you think that because of taking care of patients, you don't have time to do your own personal affairs? | 0 | 1 | 2 | 3 | 4 |
| 12. Do you think that your social life has been affected because of caring for patients? | 0 | 1 | 2 | 3 | 4 |
| 13. Have you ever considered giving up inviting friends over to your home because the patient is at home? | 0 | 1 | 2 | 3 | 4 |
| 14. Do you think that the patient merely relies on your care and regards you as the only person he/she can trust? | 0 | 1 | 2 | 3 | 4 |
| 15. Do you think that apart from your own expenses, you have no spare money available for caring for the patients? | 0 | 1 | 2 | 3 | 4 |
| 16. Do you think it's possible for you to spend more time caring for the patients? | 0 | 1 | 2 | 3 | 4 |
| 17. Do you think that since starting the care process, it has become impossible for you to live as you wish? | 0 | 1 | 2 | 3 | 4 |
| 18. Would you like to have the patient left to be taken care of by someone else? | 0 | 1 | 2 | 3 | 4 |
| 19. Do you have any situation where you're unsure of what to do regarding the patient? | 0 | 1 | 2 | 3 | 4 |
| 20. Do you think more should be done for the patients? | 0 | 1 | 2 | 3 | 4 |
| 21. Do you think you could do a better job in caring for patients? | 0 | 1 | 2 | 3 | 4 |
| 1. Overall, how do you evaluate your burden in terms of nursing? | No | Light | Moderate | Heavy | Extremely heavy |
